# Mobile Phone Passive Positioning through the Detection of Uplink Signals for Search and Rescue

**DOI:** 10.3390/s19204526

**Published:** 2019-10-17

**Authors:** Yuhui Gao, Zhongliang Deng, Yao Zhang, Shihua Sun, Zhen Li

**Affiliations:** School of Electronic Engineering, Beijing University of Post and Telecommunication, Beijing 100876, China; dengzhl@bupt.edu.cn (Z.D.); yaoo@bupt.edu.cn (Y.Z.); sshua@bupt.edu.cn (S.S.); manniu677@gmail.com (Z.L.)

**Keywords:** OFDM, LTE, preamble detect, passive positioning

## Abstract

To satisfy the service requirements of high accuracy and efficient life detection and location for search and rescue (SAR) missions after a disaster, we developed a passive positioning method to locate mobile phones by capturing the random access preamble, which can be applied to fourth-generation (4G) and even fifth-generation (5G) communication systems. We analyzed the characteristics of the random access procedure of a communication system and established a way to detect mobile phones by combining the time-difference-of-arrival (TDOA) estimation to determine the location. Then, we performed an experiment and a simulation of preamble sequence acquisition, and the results proved that the method is feasible and has high detection accuracy in high-noise conditions.

## 1. Introduction

In the face of a natural disaster, a safe, reliable, and efficient technique for carrying out search and rescue (SAR) missions is urgently needed. Recently, some new methods have been proposed; for example, unmanned aerial vehicles (UAVs) with various sensors (such as high-quality image sensors and thermal infrared sensors) are used for SAR missions [1,2,3,4], and some SAR operations are assisted by a robot team which can go deep into the ruins [5,6,7,8]. These new technologies have improved the efficiency of SAR to a certain extent, making SAR team efforts more convenient. However, these methods are expensive and do not have the ability to search in parallel and to quickly locate trapped people across a wide area at the same time. Thus, improving the efficiency of SAR missions after a disaster is still a problem worth studying. With the development of mobile Internet technology, smart phones have become an indispensable part of people’s lives, and location-based services (LBS) have broad application prospects in the Internet-of-Things (IoT) era, thus providing a new basis for SAR efforts.

Base station (BS) positioning is a service provided by mobile operators, which does not require Global Navigation Satellite Systems (GNSS) or professional positioning devices. In many countries, mobile operators are required to provide emergency services for mobile phone users to detect the location of callers for SAR. For example, the US Federal Communications Commission has established E911 regulations [9,10]. Because of the large coverage area of a BS, positioning accuracy is very limited because of the influence of channel fading and non-line-of-sight (NLOS). For complex disaster environments, the traditional BS positioning method is difficult to apply. Presently, many research institutions have been involved in studying emergency rescue systems based on mobile phone signal searches in disaster scenarios. The most representative of these efforts is the Project I-LOV in Germany, which uses Global System for Mobile Communications (GSM) signals for post-disaster relief work [11,12]. However, there are currently few studies on long term evolution (LTE). Compared to GSM, LTE has a wider bandwidth and uses orthogonal frequency division multiplexing (OFDM) and multiple-input multiple-output (MIMO) technology, which can provide a basis for high-precision positioning in disaster environments.

Various positioning methods are specified in the 3gpp protocol standard [13,14], including observed time difference of arrival positioning (OTDOA), assisted global navigation satellite system (A-GNSS), enhanced cell ID, and so forth. OTDOA is observed by the user equipment (UE) to measure the arrival time of a reference signal sent by multiple neighboring BSs and calculate the arrival time difference to obtain the location. A-GNSS uses the satellite navigation chip in the UE, which acquires satellite signals for positioning and has high positioning accuracy but is not suitable for dense urban or indoor environments. Enhanced cell ID positioning is the easiest and fastest positioning method, which determines the location of a mobile phone through accessing the cell, but has the worst positioning accuracy [15]. These methods are all active positioning, which requires the UE to capture signals and calculate the position; thus, they are not applicable to SAR missions.

To satisfy the service requirements of high accuracy and efficient life detection and location for SAR missions after a disaster, based on our previous work [16], we developed a mobile phone passive positioning method that detects the random access preamble of fourth-generation (4G) and fifth-generation (5G) communication systems. Considering the detection accuracy and computational complexity, an optimized preamble detection procedure was used and implemented with a field-programmable gate array (FPGA), and the feasibility of the method was verified by experiments. The detection accuracy under high-noise conditions was analyzed by simulation, considering that signals emitted from ruins in the post-disaster environment are too weak to be detected. The positioning method signal acquisition and position calculation are performed at the BS, so it is not necessary to obtain mobile phone identity information and resource allocation status in advance. This method can be applied to an existing commercial BS to trigger mobile phone access, and can also use a pseudo BS to induce mobile phone access if a commercial BS fails to work normally after a disaster.

The contributions of this paper are as follows:

- A passive positioning method to locate 4G mobile phones by detecting uplink signals is developed, which can be utilized to quickly locate people buried in shallow ruins across a wide area for SAR missions.
- An analysis of the random access process and an introduction to the positioning model are given. According to the analysis, the signal detection procedure is presented and implemented with FPGA.
- Simulation and analysis of the detection procedure are conducted and the results show that the positioning method is feasible and has high detection accuracy in high-noise conditions in the post-disaster environment.

This paper is organized as follows: Section 1 gives an introduction to emergency rescue systems based on mobile phones and the existing mobile communication system positioning method. Section 2 analyzes the random access procedure of the mobile communication system and the preamble sequence used for positioning. Section 3 elaborates the positioning model and signal detection procedure. Section 4 illustrates the experimental process and the analysis of the results. Finally, the conclusions are given in Section 5.

## 2. Random Access

In 4G and 5G communication systems, random access is a procedure in which a UE accesses a BS [17,18]. The random access procedure undertakes the function of establishing a wireless link through the initial access and uplink synchronization, authenticating the identity of the UE and applying for uplink resources, which makes it the first choice for signal detection for disaster relief.

### 2.1. Random Access Procedure

The random access procedure is implemented by a physical random access channel (PRACH) and is divided into contention-based random access (CBRA) and contention-free random access (CFRA). The former is initiated by the UE, and the latter is performed by the UE according to the indication of the BS.

**Figure 1:**
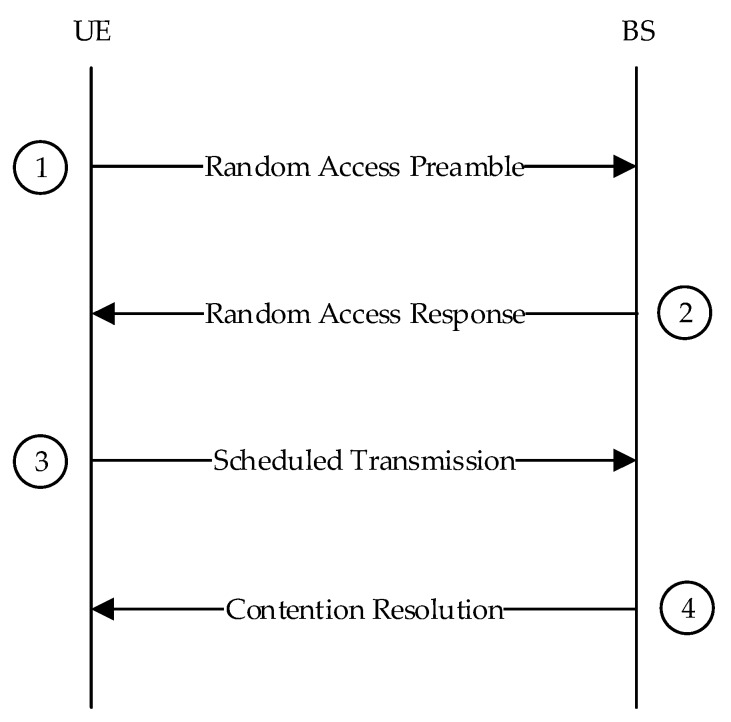
Contention-based random access procedure.

The CBRA procedures consist of four steps, as shown in Figure 1.

Random access preamble on random access channel (RACH) in uplink. The UE sends a random access preamble to the BS.

Then, random access response (RAR) is generated by medium access control (MAC) on Downlink Shared Channel (DL-SCH). The BS sends a RAR, including a time advance command according to the estimated time in the previous step, Uplink (UL) grant allocating uplink resources for the UE, and a temporary cell radio network temporary identifier (TC-RNTI).

First scheduled UL transmission on Uplink Shared Channel (UL-SCH). The UE obtains the uplink synchronization according to the time advance command and sends a radio resource control (RRC) connection request, which includes the temporary identity information and the contention resolution identifier in the uplink resource allocated by the BS.

Contention resolution on Downlink (DL). The BS sends a corresponding signal to the UE to resolve the random access collision.

**Figure 2:**
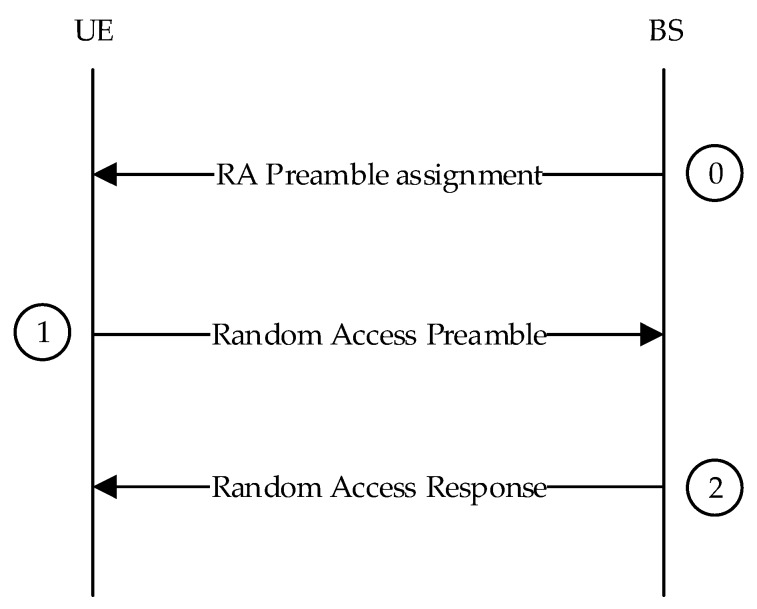
Contention-free random access procedure.

The CFRA procedures consist of three steps, as shown in Figure 2.

Random access preamble assignment via dedicated signaling in DL. The BS allocates a preamble sequence and a PRACH access resource to the UE through a dedicated signal.

Random access preamble on RACH in uplink. The UE sends a random access preamble to the BS.

RAR on DL-SCH. The BS sends a RAR.

The UE positioning method based on random access preamble detection is performed by capturing the random access preamble sent in step 1 of the random access procedure and utilizing the follow-up process to implement identity recognition.

### 2.2. Random Access Preamble

**Figure 3:**
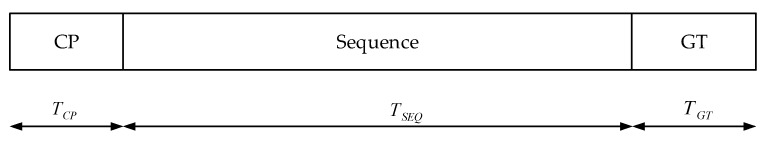
PRACH channel format.

As shown in Figure 3, the random access preamble consists of a cyclic prefix (CP) of length $T_{CP}$, a preamble sequence of length $T_{SEQ}$, and a guard time (GT) during which nothing is transmitted [19]. The parameters change depending on the frame structure and random access configuration. Different cell sizes determine the different channel formats that are used. There is a delay when random access signals propagate from UE to BS within the maximum cell sizes. When the propagation path is less than the maximum cell sizes, the position where the sample point of the received preamble signal ends is always in the GT, thus ensuring complete reception of the signal. A CP can effectively reduce the effects of multipath delay spread, intercarrier interference, and intersymbol interference.

For a preamble format of 0–3, the preamble sequence consists of a series of Zadoff–Chu sequences with a length of 839, which have strong auto/cross-correlation and a low peak-to-average power ratio. Each cell has 64 available preamble sequences, which are generated by the cyclic shift of the root sequence. Different cell sizes also determine the number of cyclic shifts. When a root sequence is not sufficient to generate all the preamble sequences, another root sequence is used. Adjacent cells use different preamble sequences to prevent collisions.

According to [19,20], the *u*th root Zadoff–Chu sequence is defined by

(1) $$x_{u}\left(n\right)=\exp\left(-j\frac{\pi un\left(n+1\right)}{N_{ZC}}\right),\;n=0,1,\ldots ,N_{zc}-1,$$

where $N_{zc}=839$ or $N_{zc}=139$ depending on the PRACH preamble format. Assuming that the trapped person is motionless, we would only consider unrestricted set situations. From the *u*th root Zadoff–Chu sequence, preamble sequences with zero correlation zones of length $N_{zc}-1$ are defined by cyclic shifts according to

(2) $$x_{u,v}\left(n\right)=x_{u}\left(\left(n+vN_{cs}\right)modN_{zc}\right),\;n=0,1,\ldots ,N_{zc}-1,$$

where $N_{cs}$ is the cyclic shift value, and $v=0,1,\ldots ,\lfloor N_{ZC}/N_{cs}\rfloor -1$. The time-continuous random access signal s(t) is defined by

(3) s(t)=βPRACH∑k=0NZC−1∑n=0NZC−1xu,v(n)exp(−j2πnkNZC)exp(j2π(k+φ+K(k0+12)ΔfRA(t−Tcp))),

where $0\leq t<T_{SEQ}+T_{CP}$, $\beta_{PRACH}$ is an amplitude scaling factor in order to conform to the transmit power, $k_{0}$ controls the location in the frequency domain, $K=\Delta f/\Delta f_{RA}$, Δf is the subcarrier spacing, and $\Delta f_{RA}$ is the subcarrier spacing of RA. φ is a fixed offset determining the frequency domain location of the random access preamble within the physical resource blocks.

**Figure 4:**
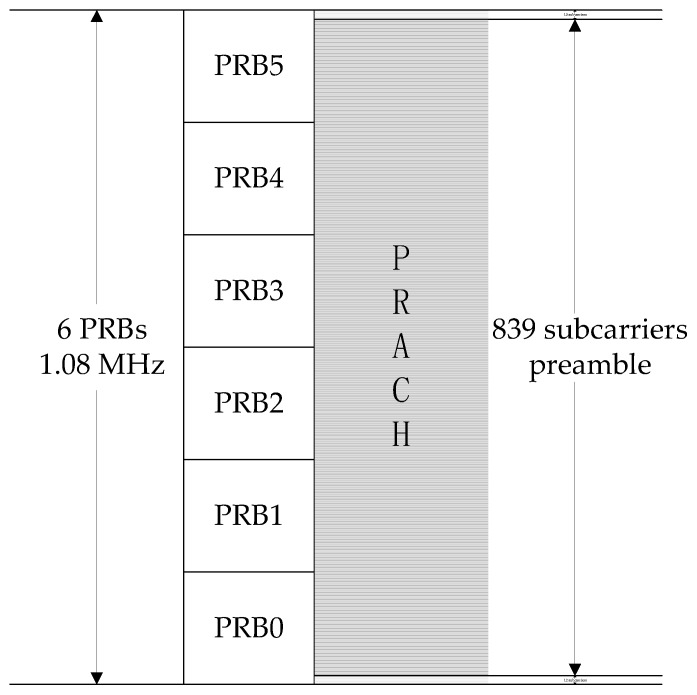
Physical random access channel format.

According to the function, the preamble sequence map is on 839 subcarriers with 1.25 kHz subcarrier spacing in the frequency domain of PRACH. As shown in Figure 4, there are 12 and 13 subcarrier spacings used as protection bandwidth at both ends, respectively. The 864 subcarriers occupy a bandwidth of 1.08 MHz, which is equivalent to six physical resource blocks (PRBs). In a 20 MHz bandwidth LTE system, there are generally 100 PRBs, and the PRACH is located in 6 PRBs at a specific location.

The index of the root sequence and the cyclic shift value of the preamble generation are broadcasted by the BS to the mobile phone. After the downlink synchronization, the mobile phone randomly selects a preamble sequence according to the broadcast to transmit the preamble in the specific subframe and resource block.

## 3. Positioning Method and Signal Detection

In [21,22], they investigated cooperative positioning using uplink measurements in LTE cellular networks called uplink TDOA (UTDOA) measurement, which requires a BS to be equipped with a location measurement unit and UE connected. Similar to UTDOA, our method achieves a three-dimensional positioning function by combining at least four detecting devices, which can be four BSs or one pseudo BS cooperating with three receivers, and requires each device to have high time consistency and a precise reference position. In the following discussions, it is assumed that four BSs are used for positioning.

### 3.1. Positioning Method

The main process of our positioning method is as follows: First, the BS induces the mobile phone to initiate a random access procedure and transmit a random access preamble at a specific time–frequency location. Second, the four BSs measure the arrival time of the signal and calculate the physical position of the UE by TDOA and an equation-solving method. Finally, the preamble sequence selected by the UE is identified and the identity of the UE is determined according to the time–frequency location of the signal and the subsequent access procedure.

**Figure 5:**
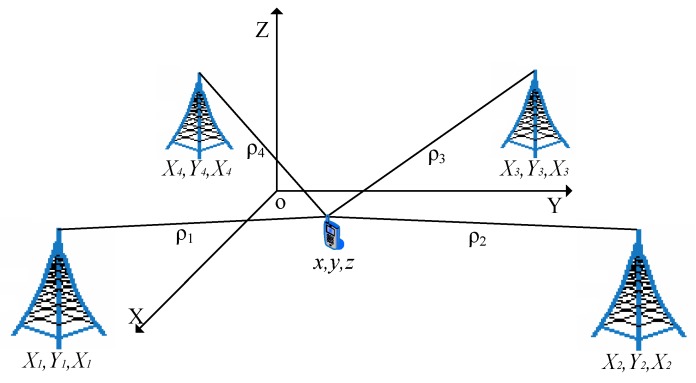
Four BSs joint positioning.

As shown in Figure 5, the UE is located in ${\left(x,y,z\right)}^{T}$, and the BSs are located at ${\left(X_{i},Y_{i},Z_{i}\right)}^{T}$ for i=1,2,3,4. The signal time is $t_{u}$, the time of arrival is $t_{i}$, the propagation time $\Delta t_{i}\;$= $t_{i}-t_{u}$, and C is the propagation speed of the radio wave. It is assumed that the four positioning apparatus have the same clock and no relative time error. At the same time, the distance $\rho_{i}$ between UE and BS is measured by different BSs. The positioning solution equations can be listed as follows:

(4) $$\rho_{i}=C\Delta t_{i}=\sqrt{{\left(X_{i}-x\right)}^{2}+{\left(Y_{i}-y\right)}^{2}+{\left(Z_{i}-z\right)}^{2}}+\varepsilon_{i},$$

where $\varepsilon_{i}$ is the measurement error. Taking the distance between the first BS and the mobile phone as the reference distance, the difference of the distance between the *i*th BS and the reference BS can be expressed as

(5) $$\begin{array}{ll} & {\tilde{\rho }}_{i,1}=C\Delta t_{i}-C\Delta t_{1}\\ = & C\left(t_{i}-t_{1}\right)\\ = & =\sqrt{{\left(X_{i}-x\right)}^{2}+{\left(Y_{i}-y\right)}^{2}+{\left(Z_{i}-z\right)}^{2}}-\sqrt{{\left(X_{1}-x\right)}^{2}+{\left(Y_{1}-y\right)}^{2}+{\left(Z_{1}-z\right)}^{2}}+\varepsilon_{i}-\varepsilon_{1}. \end{array}$$

Converted to a matrix form, this can be expressed as

(6) $$\tilde{\rho }=\rho +\varepsilon ,$$

where ρ is the true value of the distance difference between the reference BS and other BSs to the mobile phone, $\tilde{\rho }$ is the measured value, and ε is the measurement error. The upper nonlinear equations are solved by the least-squares algorithm, and the mobile phone positions ${\left(x,y,z\right)}^{T}$ are finally estimated.

The methods of the BS’s position detection, time synchronization, and position solving which affect the positioning accuracy are similar to the conventional methods and are not described in this paper. The process of capturing the preamble sequence for correlation analysis is discussed below.

### 3.2. Preamble Detection

The root sequence, the value of cyclic shift, and the time–frequency position are broadcasted as part of the system information from the BS, so it is only necessary to detect at the location where the preamble may exist. If the cell uses multiple root sequences, each root sequence needs to be detected separately. Since the preamble arrives at each BS with different delays, the correlation peak positions obtained by correlation analysis of each BS are different. Therefore, after detecting the preamble, the arrival time of the signal can be obtained by correlation analysis combining the frame number, subframe number, and timestamp.

In order to obtain a more accurate arrival time, the signal needs to be sampled with a larger sampling frequency. Because of severe signal attenuation in a post-disaster environment, it is necessary to perform fast Fourier transform (FFT) with as many sampling points as possible to suppress the noise effects. For the inverse FFT (IFFT), 1024 points are sufficient for detection, as the preamble sequence has only 839 points in the frequency domain, but all points are needed to obtain the accurate arrival time. If a random access procedure does not exist, this results in redundant calculations. Therefore, preamble sequence detection is performed first using 1024-point IFFT to improve the detection speed. When it is judged that the preamble sequence exists, large-point IFFT is used for correlation analysis to improve the arrival time measurement accuracy. Preamble detection for the positioning algorithm is described in Algorithm 1 and the brief implementation process is as follows:

According to the parameters of the BS, the uplink channel is downconverted, filtered, and sampled to obtain time domain discrete signals. These processes are implemented by the radio frequency front end.

Intercept sampling points where the preamble may exist as *s* and append a timestamp; then, FFT is performed to obtain a frequency domain sequence S(n).

The 839 points of the frequency domain sequence which is mapped by the preamble sequence are intercepted as $S^{\prime }\left(n\right)\;0<n\leq 839$, multiplication is performed with the conjugation of the local root sequence Xu, and the result is temporarily saved for subsequent processing.

Then, fill the end of the result sequence with zeros and perform 1024-point IFFT to obtain a time domain correlation sequence. The obtained time domain correlation sequence value is modulo squared, and then sequence peak detection is performed.

If the peak value is greater than the decision threshold, there is random access and the preamble sequence index used for random access is determined; if the peak value is smaller than the decision threshold, there is no random access.

If random access is detected, read temporarily saved conjugate point multiplication results and perform large-point IFFT. The relevant peak position is determined to obtain the signal rough arrival time, and then the phase difference is calculated to achieve fine measurement.
**Algorithm 1** Preamble detection for positioning 1: **Procedure Pdfp**(s, Xu, threadhold, k, N) 2: S←FFT(s) 3: S’(n)←S(n + k) n = 1,2…,839 4: p(n)←S’(n) × Xu^∗^(n) n = 1,2…,839 5: r←IFFP(p, 1024) 6: **if** max(r) > threadhold **then** 7:  r←IFFP(p, N) 8:  **for** n←1 to N **do** 9:   **if**
$\bar{r\left(n-1\right)}<\bar{r\left(n\right)}\;\&\&\;\bar{r\left(n+1\right)}<\bar{r\left(n\right)}\;\&\&\;\bar{r\left(n\right)}>\mathrm{threadhold}$ 10:    out←[n,r(n)] 11:   **end if** 12:  **end for** 13:  **return** out 14: **end if** 15: **end procedure**


**Figure 6:**
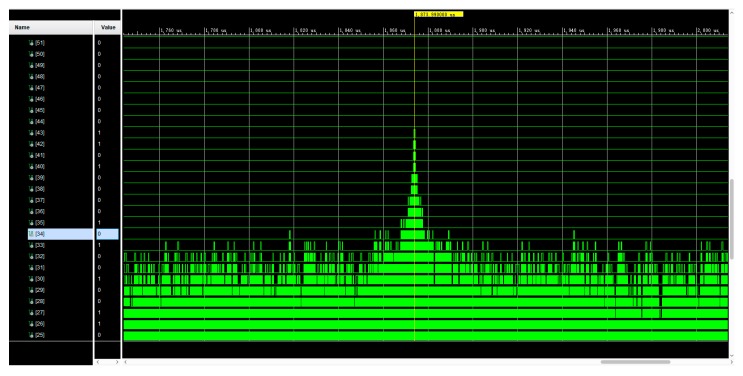
Correlation peak in simulation of preamble detection.

We implemented the process of preamble detection for positioning with Analog Devices AD-FMComms3-EBZ and Xilinx VC707 FPGA boards and performed simulations using Xilinx Vivado 2019.1 (San Jose, California, U.S.). The results of the correlation are shown in Figure 6.

## 4. Experiment and Simulation

The test results of the preamble detection are presented in this section. In order to test the feasibility and accuracy of the method, we built a BS to induce a mobile phone to complete the random access procedure, captured the preamble signal sent by the mobile phone during random access, and performed simulations with Matlab 2017b.

### 4.1. Preamble Detection Experiment

We built a pseudo BS by using a software-defined radio (SDR) platform which can stimulate 4G mobile phones to start the random access procedure. Uplink and downlink transmissions are separated by frequency division-duplex (FDD) and other basic BS parameters are given in the Table 1. The sampling rate of the receiver was 30.72 MHz, and the bandwidth of the receiver was 18 MHz.

**Figure 7:**
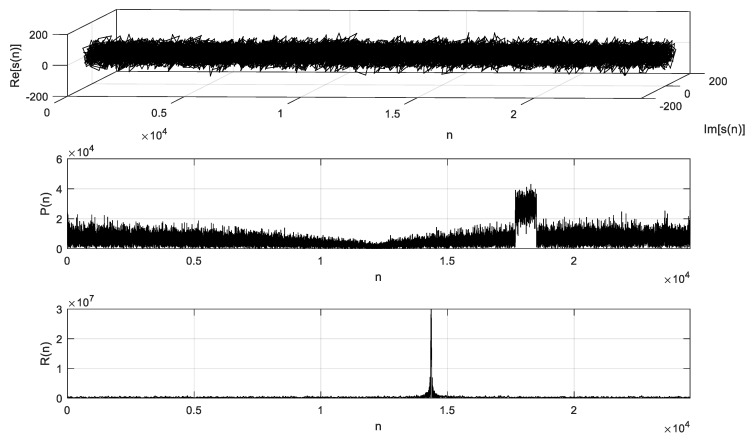
Time domain, power spectrum, and the corresponding correlation peak.

The data of sample points of one subframe were saved once a preamble signal was detected. As the sampling frequency was 30.72 MHz, a total of 30,720 sampling points were obtained for 1 ms as a subframe, in which there were 3168 sampling points of the cyclic prefix and 24,576 sample points of the preamble sequence; the remaining sample points were guard time. After CP removal processing of the sample points, the remaining 24,576 points were used for FFT and correlation analysis. The time domain, power spectrum, and the corresponding correlation peak are shown in Figure 7.

### 4.2. Preamble Correlation

#### 4.2.1. Single Preamble Correlation

**Figure 8:**
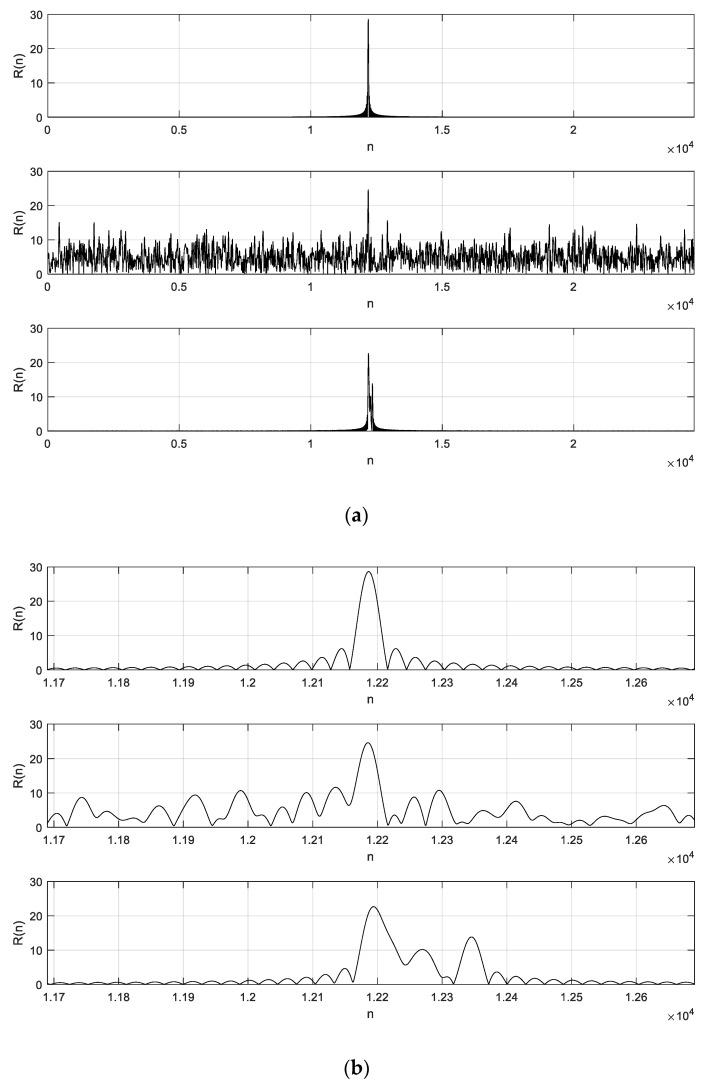
Preamble sequence correlation peak: (**a**) all points and (**b**) part of the points.

As shown in Figure 8, we performed correlation detection simulation of a single preamble sequence in three cases: direct autocorrelation, autocorrelation superimposing additive white Gaussian noise (AWGN), and autocorrelation through a multipath fading channel.

It can be seen from Figure 8 that a sharp correlation peak was obtained after the correlation analysis, and the resolution reached one sampling point. When the sampling frequency was 30.72 MHz, the time interval of each sampling point was 32.55 ns. Considering the electromagnetic wave propagation speed of $3\;\times \;10^{8}\;m/s$, the distance interval of adjacent sampling points was about 10 m, so the measurement resolution was 10 m.

**Figure 9:**
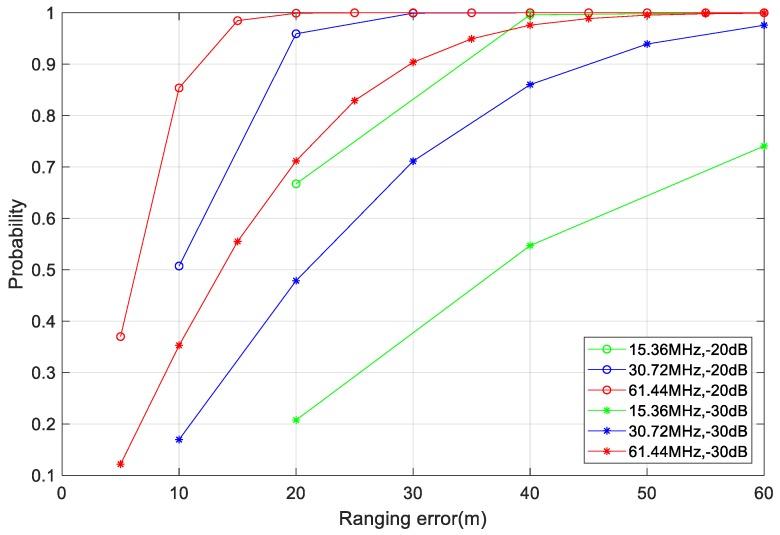
Probability of ranging error within different distances.

Figure 9 shows the probability of being within different ranging errors at sampling rates of 15.36, 30.72, and 61.44 MHz under −20 and −30 dB signal-to-noise ratio (SNR). When the SNR was −20 dB, the probability of ranging error within 20 m was more than 95%, so distance measurement accuracy within 20 m could be achieved. Although the ranging error was greatly increased when the SNR was −30 dB at the same sampling rate, the signal was still detected. Using a high sampling rate allowed for higher detection accuracy and greater noise immunity than using a low sampling rate, but it also had higher requirements for hardware processing power.

#### 4.2.2. Multiple Preamble Correlation

The actual random access procedure may have multiple UEs using the same PRACH resource in the same subframe, and there may even be multiple UEs that choose the same preamble sequence, which may cause ranging error.

**Figure 10:**
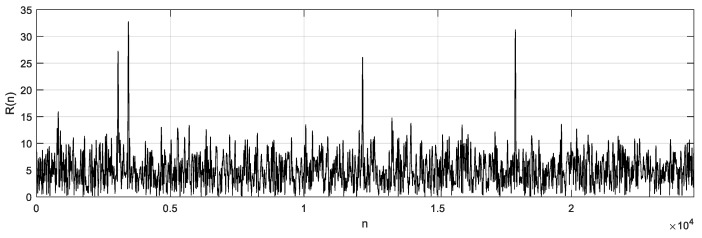
Four different preamble correlations.

As shown in Figure 10, we performed a correlation detection simulation of four different preambles superimposing AWGN. We could clearly distinguish the position of the four correlation peaks, and the probability of ranging error was the same as that of a single preamble.

**Figure 11:**
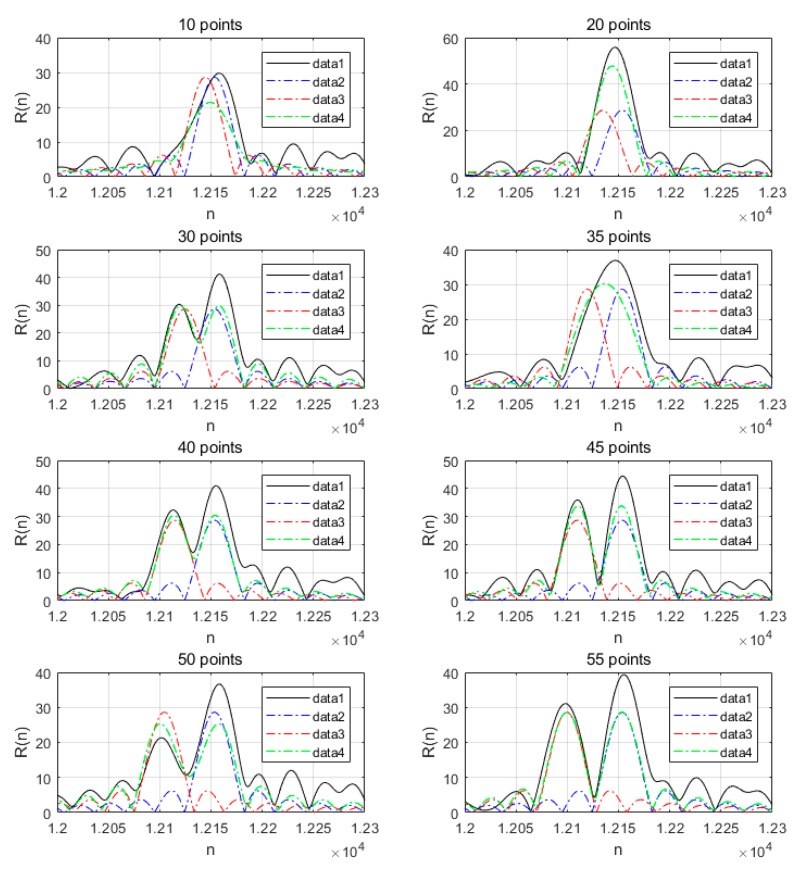
Two identical preamble correlations with different distance intervals.

Figure 11 illustrates the correlation detection simulation in partial cases, where there were two mobile phones using the same preamble sequence to initiate random access with different distance intervals, which means that contention of random access occurred. The sampling rate was 30.72 MHz and the distance of one sampling point was about 10 m. Data1 is the correlation result when two mobile phones simultaneously accessed superimposing AWGN; data2 and data3 are the correlation results when the two mobile phones accessed separately under ideal conditions; data4 is the correlation result when two mobile phones accessed simultaneously under ideal conditions. When the distance between the two phones was small, only one peak was detected. Even if two peaks are detected, there will be a large ranging error. The mutual influence of the two peaks will be reduced to a minimum when the distance reaches 55 points, which requires that the distance difference between the two mobile phones to the same BS be greater than 550 m. Therefore, it is best to avoid contentions of random access.

## 5. Conclusions

In this work, a passive positioning method to locate 4G mobile phones by capturing the random access preamble was developed, which is intended to be used for SAR missions after a disaster. In post-disaster ruins, signal propagation has serious NLOS and noise effects, which greatly reduce the positioning accuracy and communication quality. To prove the feasibility and accuracy of the method, experiments and simulations of preamble detection were conducted in high-noise conditions. According to the simulation results, although the ranging error became larger when contention occurred, it could be avoided by increasing the PRACH resources and controlling the BS transmit power. In other cases, the preamble sequence detection maintained the probability of ranging error within 20 m of more than 95% at sampling rates of 30.72 MHz with −20 dB SNR, which proves that the method is feasible. Reducing measuring mistakes and time synchronization errors and improving the detection in NLOS situations are still problems that should be studied.

## Figures and Tables

**Table 1:** BS parameters.

Parameter	Value
Frame type	FDD
Bandwidth	20 MHz
Uplink frequency	2.565 GHz
Preamble format	0
Root index	0
PRACH configuration index	0
$\;N_{cs}$ value	13
Logical root sequence number	0
PRACH high speed	disable
